# Towards Automated Eye Diagnosis: An Improved Retinal Vessel Segmentation Framework Using Ensemble Block Matching 3D Filter

**DOI:** 10.3390/diagnostics11010114

**Published:** 2021-01-12

**Authors:** Khuram Naveed, Faizan Daud, Hussain Ahmad Madni, Mohammad A.U. Khan, Tariq M. Khan, Syed Saud Naqvi

**Affiliations:** 1 Department of Electrical and Computer Engineering, COMSATS University Islamabad (CUI), Islamabad 45550, Pakistan; hamadnig@gmail.com (H.A.M.); syedsaudnaqvi@gmail.com (S.S.N.); 2 School of Information Technology, Faculty of Science Engineering & Built Environment, Deakin University, Locked Bag 20000, Geelong, VIC 3220, Australia; faizan.abdullah@outlook.com; 3 Department of Electrical Engineering, Namal Institute, Mianwali, Namal 42200, Pakistan; asmat.khan@namal.edu.pk

**Keywords:** retina, Diabetic Retinopathy (DR), Vascular Endothelial Growth Factor (VEGF), Block Matching 3D (BM3D), speckle noise

## Abstract

Automated detection of vision threatening eye disease based on high resolution retinal fundus images requires accurate segmentation of the blood vessels. In this regard, detection and segmentation of finer vessels, which are obscured by a considerable degree of noise and poor illumination, is particularly challenging. These noises include (systematic) additive noise and multiplicative (speckle) noise, which arise due to various practical limitations of the fundus imaging systems. To address this inherent issue, we present an efficient unsupervised vessel segmentation strategy as a step towards accurate classification of eye diseases from the noisy fundus images. To that end, an ensemble block matching 3D (BM3D) speckle filter is proposed for removal of unwanted noise leading to improved detection. The BM3D-speckle filter, despite its ability to recover finer details (i.e., vessels in fundus images), yields a pattern of checkerboard artifacts in the aftermath of multiplicative (speckle) noise removal. These artifacts are generally ignored in the case of satellite images; however, in the case of fundus images, these artifacts have a degenerating effect on the segmentation or detection of fine vessels. To counter that, an ensemble of BM3D-speckle filter is proposed to suppress these artifacts while further sharpening the recovered vessels. This is subsequently used to devise an improved unsupervised segmentation strategy that can detect fine vessels even in the presence of dominant noise and yields an overall much improved accuracy. Testing was carried out on three publicly available databases namely Structured Analysis of the Retina (STARE), Digital Retinal Images for Vessel Extraction (DRIVE) and CHASE_DB1. We have achieved a sensitivity of 82.88, 81.41 and 82.03 on DRIVE, SATARE, and CHASE_DB1, respectively. The accuracy is also boosted to 95.41, 95.70 and 95.61 on DRIVE, SATARE, and CHASE_DB1, respectively. The performance of the proposed methods on images with pathologies was observed to be more convincing than the performance of similar state-of-the-art methods.

## 1. Introduction

Analysis of biomedical images is one of the growing research fields. It is related to the study and analysis of digital images based on image processing techniques using computational tools that help in the analysis of clinical problems [1,2,3]. Recently, rapid progress in the research domain of biomedical image processing has proven significantly important as it reduces the use of invasive approaches for diagnosis purposes.

This research is based on the analysis of retinal fundus images for the diagnosis of eye disease using computerized techniques. The retina is present in the interior surface of the eye, which possesses photoreceptors that are the cells sensitive to light. They convert light into neural signals that are taken to the brain via optic nerves. To visualize the retina, a retinal image (also called fundus image) can be obtained by a fundus camera system (retinal microscope), which is typically used to capture retinal images. The retinal image comprises important diagnostic information that helps to identify healthy or unhealthy retina. Retinal images have been commonly used to diagnose vascular and non-vascular pathology in the medical world [4]. Retinal blood vessels can be used to diagnose different eye diseases as well as other diseases like diabetic retinopathy, glaucoma, and hypertension. Moreover, each individual has a distinct network of blood vessels, so they can also be used for biometric identification. The retinal blood vessel structure is a very complex network and an efficient algorithm is required to detect it automatically. Diabetic retinopathy (DR) is an eye-related condition which is the primary cause of blindness. It is a diabetes mellitus complication and is caused by retinal vasculature damage. Patients only know this silent disorder when they have sight problems. However, this happens when retinal changes have developed to a point where there is a higher risk of vision loss and treatment is complicated [5]. There are two types of DR, Non-Proliferative Diabetic Retinopathy (NPDR) and Proliferative Diabetic Retinopathy (PDR). DR is normally identified by eye scanning [5]. Early-stage diagnosis of eye-related disorders has a significant effect on keeping the patient from losing vision [6].

Various pathological conditions occur due to leakage of blood vessels like hemorrhages, lipid and hard exudates (in which protein fluid leaks or deposits), and a capillary blockage (called cotton wool spots). It may also cause swellings in the tiny capillary wall known as microaneurysms, which is a solid indication of diabetic retinopathy [7]. PDR is an advanced stage of DR that causes retinal ischemia, which affects the blood flow into and out of the retina and results in poor nourishment of the retina. The unnourished retinal areas transmit the nourishment signals for the supply of oxygen in terms of growth factors like Vascular Endothelial Growth Factor (VEGF). It leads to the growth of new blood vessels in the retina, known as Neovascularization [8]. The new blood vessels that have formed in this way are fragile. They may grow on the retinal surface or sometimes on the optical structure called the iris. These blood vessels can break down or leak and cause fibro-vascular proliferation. When these vessels grow on the iris [9], they may block the filter that drains the fluid from the eye, due to which the pressure rises inside the eye that may result in neurovascular glaucoma causing blindness [2,10].

Excessive sugar in the blood for a long time may cause the blockage of the tiny blood vessels that nourish the retina, resulting in cutting off the supply of blood to the retina. Eventually, new blood vessels are grown in the eye that are not properly developed and cause blood leakage. People with diabetes can suffer from DR. Other factors that are responsible for DR are [11]:

- Long duration of diabetes,
- Improper blood sugar level control,
- High blood pressure and cholesterol,
- Use of tobacco.

Hence, understanding of the structural attributes of blood vessels in the retina is key to addressing this problem. Therefore, vessel segmentation is at the very core of the computerized methods for automated detection of eye disease(s). Specifically, segmentation methods employ length, width, orientation and branches as main attributes when tracking and segmenting vessels from within the fundus image. In this regard, supervised methods are known to yield comparatively better performance metrics owing to the complex multistage procedure. Specifically, supervised methods extract feature vectors from a large set of data to train the classifiers (i.e., support vector machines or neural networks), which are eventually used to make decisions based on the learned features [11]. The main criticism of these methods is the high computational expense and certain degree of sophistication involved. Nevertheless, the availability of well curated datasets (specific to the disease) is another major challenge limiting the use of these methods in practice.

On the contrary, unsupervised methods estimate the health of blood vessels through filter responses, vessel tracking methods or model based approaches. The use of traditional image processing techniques requires much less (computational and data) resources. Hence, these classical methods do not suffer from the practical constraints faced by the supervised methods. That makes them very much relevant in practice today despite their comparatively lower performance. In fact, it only makes the case for the improvement in the performance of the classical vessel segmentation methods by alleviating their inherent disadvantages. That is precisely the motivation behind our work where we address the challenge posed by noise in detection of vessels.

Noise is introduced in the fundus image during the complex acquisition process that also causes poor image contrast and anatomical variability of the vessel. In order to solve these issues of noise and artifacts, the method of segmentation of the retinal vessel usually consists of two stages: preprocessing and vessel extraction. Many techniques were focused solely on the second phase [12,13], that is not optimal due to the obtained simulation results indicate false-positive pixels induced by sensor noise present in the image. Hence, it is necessary to perform preprocessing a priori in order to extract relevant information from the retinal image. Here, the goal is to enhance visual appearance of objects by reducing noise such that small vessels are not harmed. Mainly, preprocessing stage employ filtering to increase the accuracy of the vessel segmentation.

Various sounds and unpredictable fluctuations in frequency levels also distort the recorded pictures. It is widely understood that fundud images are corrupted by additive noise that is commonly known as Gaussian noise, multiplicative (also known as speckle) noise and Shot noise (famously known as Poisson noise). There are many methods in the literature, mainly aimed at removing noise from images. Such filtering methods can be classified into two groups, linear and non-linear. Linear filters, i.e., Gaussian or Wiener filters, are particularly good for the reduction of Gaussian noise and, in some situations, for non-conventional non-Gaussian noises. Within linear filters, pixel values are updated by assigning weights to neighboring pixels and computing the weighted sum. Owing to that linear filters suffer from blurring and over smoothing that reduces the image quality and loss of sharp discontinuities.

On the other hand, non-linear filters have proven to more useful when reducing noise without losing the discontinuities. Anisotropic Diffusion of Perona and Malik (PMAD) is the most efficient non-linear filter that employs a multiscale framework for edge detection and noise smoothing [14]. Owing to its efficacy multiple extensions of this filter have been develop for addressing the issue of speckle noise within monochrome images, for example, Speckle-Reducing Anisotropic Diffusion (SRAD) [15], Flux-Based Anisotropic Diffusion (FBAD) [16] and Detail-Preserving Anisotropic Diffusion (DPAD) [17]. These approaches require advance definition noise characteristics which are assumed to be consistent across the entire image. That is not true in reality. Therefore, these methods fail to provide optimal performance for images attenuated the noise with unknown models. That is because the selection of accurate noise is absolutely essential to improve the efficiency of these filtering algorithms.

Keeping in view the aforementioned limitations, more evolved denoising methods employ filtering in the wavelet domain, for instance, [18,19,20,21,22,23,24]. Although these methods require prior knowledge of the noise model, their performance is not affected by the non-stationarity of the input image. Therefore, wavelet-based methods dominate the literature on image denoising mainly due to their low computational cost and state-of-the-art performance. Another class of methods exploits the redundancy of image features in the spatial domain where similar image patches (i.e., a group of neighboring pixels) are processed together to suppress noise [25,26,27,28]. These methods, though computationally expensive, significantly reduce noise but in the process also smooth out edges within an image, which is what limits their applications.

These two techniques in conjunction give birth to a class of hybrid methods, which combine the benefits of redundancy in the spatial domain and effectiveness of noise removal in a wavelet or other transform domains, for example, block matching 3D (BM3D) filter [29]. The *BM3D* filter, originally designed to tackle the additive Gaussian noise, is widely accepted as the gold standard noise removal method owing to its sophisticated multi-step procedure. To benefit from this superior architecture, an extension of the *BM3D* filter is designed to address the multiplicative speckle noise in [30]. This speckle adapted *BM3D* (S-BM3D) filter retains the structure of the original *BM3D* filter while adapting each step for addressing the speckle noise case. The efficacy of the *BM3D* filters lies in their ability to preserve edges and other image features while effectively minimizing noise. That makes them suitable for denoising the retinal fundus images where the objective is to do away with noise without losing the smaller vessels.

The challenge in unsupervised detectors is that noise in fundus images obscures smaller vessels that eludes the computerized detection of these vessels contributing to reduced efficiency [31,32]. This issue necessitates the removal of noise for improved vessel detection and segmentation [33]. In this regard, the availability of such an effective set of noise removal methods presents an exciting opportunity to incorporate these state-of-the-art denoisers within the preprocessing block of the computerized detectors.

The main criticism of the use of denoisers for restoring the fundus image is their inability to address both the additive and multiplicative noise cases simultaneously. Alternatively, a suitable approach may be to figure out the dominant noise among the two and address that case only. That is expected to yield reasonable improvement. In this regard, it can be argued that speckle, owing to its multiplicative nature and dense concentration, dominantly impacts the structural details compared to the effect caused by the systematic additive noise. Hence, prioritizing the removal of speckle patterns from the retinal fundus image can significantly improve the quality of fundus images. This is demonstrated in a recent approach in [34] whereby a state-of-the-art speckle denoiser namely probabilistic patch based (PPB) denoiser [26] is used to improve the performance of an unsupervised retinal vessel segmentation framework. This scheme separately detects small and large vessels whereby PPB denoiser was essentially used to improve the detection of large vessels.

Another motivation behind the use of a state-of-the-art speckling denoising method (e.g., S-BM3D) on retinal fundus images is that it significantly improves the contrast of the input image. Since speckle adversely effects the contrast of the input image, its removal naturally restores the contrast between the image features. This particular advantage is relevant in fundus images because these are known to suffer from low contrast issues of their own (due to poor illumination problems). Consequently, the built-in contrast improvement mechanism in despeckling methods implicitly addresses another core problem in vessels segmentation from fundus images.

Inspired by this, we present an enhanced segmentation strategy based on multiscale line detectors and a customized denoising framework for fundus images. The proposed denoising framework employs an ensemble of S-BM3D filters to effectively minimize noise without any significant loss of blood vessels in the image. However, the problem with the S-BM3D filter is that it yields dense checker board artifacts, which are ignored in satellite images due to their less sensitive applications. However, in the case of fundus images, these artifacts distort edges of vessels thus presenting a practical challenge in detection of smaller vessels. To address this issue, we propose an ensemble S-BM3D filter that minimizes the artifacts (caused by S-BM3D) while ensuring the retention of finer vessels. This enables segmentation of tiny vessels that were originally obscured by noise and artifacts. The main contributions of our work are listed below:

1. A novel ensemble filtering framework is proposed based on a gold standard S-BM3D denoiser that facilitates the detection of finer vessels, originally obscured/distorted by speckle patterns. This customized ensemble filtering framework is specifically designed to address the inherent issue of checkerboard artifacts within the S-BM3D method. That paves the way for its use in sensitive applications including the retinal vessel segmentation studied in this paper. Specifically, we have designed a customized ensemble averaging filter that tunes S-BM3D at varying parameters to control the trade off between the artifacts and the quality of vessels. That leads to different denoised images with varying artifact levels and captured details (vessels). Now, ensemble averaging these resulting images intelligently mitigates artifacts without any significant loss to edges of tiny vessels. Here, it is important to mention that our proposed ensemble averaging approach is significantly different from the standard ensemble averaging filters, which merely averages the shifted denoised images to smooth out artifacts [35,36,37]. That results in the loss of important image details along with the removal of undesired artifacts. On the contrary, our approach ensures retention of finer details due to the customized ensemble averaging framework, which is a novel contribution of this work.
2. An improved vessel segmentation framework is presented by employing the proposed ensemble S-BM3D (ES-BM3D) denoiser in the preprocessing pipeline. Specifically, we present two distinct frameworks based on each of the multiscale line detector and Frangi filter for vessel segmentation. The highlight of the proposed framework is that it is capable of segmenting vessels in presence of noise owing to the ability of the proposed denoiser to remove noise without any significant loss of vessels in the denoised fundus images. This enables the detection and segmentation of additional tiny/smaller vessels (otherwise obscured by noise) using our framework.

It is pertinent to mention that the proposed segmentation strategy employs a significantly different strategy compared to [34], which employs a denoiser to extract large vessels and smooth out small vessels and noise. As a complete contrast to that, the proposed methodology aims to uncover the tiny vessels by minimizing noise using the proposed denoiser. That allows detection of additional tiny vessels. Consequently, our vessel segmentation framework offers much improved accuracy for computerized eye disease detectors compared to the state-of-the-art methods. In this regard, we validate the efficacy of the method on three publicly available databases namely Structured Analysis of the Retina (STARE), Digital Retinal Images for Vessel Extraction (DRIVE) and CHASE_DB1.

## 2. Literature Review

A wide range of methods for image denoising has been provided in the past few years which can be essentially categorized into two conventional smoothing filters and non-conventional techniques for edge detection. Classical or conventional methods are generally based on Gaussian model and may lose the edges due to oversmoothing, e.g., [38,39,40,41]. On contrary, edge detection methods estimate prominent features while denoising for retention opd edges and corners [14,25,42,43]. Gaussian-based filtering methods are frequently used in the analysis of medical images. However, as Gaussian filter weights rely on the distance between pixel locations, these methods may end up missing prominent features which may cause blurring. That can eventually lead to difficulties vessel detection from the retinal images. Edge-preserving techniques have been suggested to resolve the loss of edges and corners. For instance, the anisotropic diffusion filter [14] and the weighted least-square filter [43] try to smooth images while maintaining corners based on gradient measurement. The non-local means filter [25] computes the filtered outcome, focusing on the resemblance of intensity and position of the pixel in the neighborhoods. BLF is differentiated by its edge-preservation capability that is because of the kernels capturing spatial characteristics and the range properties in combination. Therefore, their performance at each pixel is based on the distance across pixels and their frequency differences [42].

Because of several of its strengths, BLF is perhaps the most commonly used method for edge-preservation while denoising. First, BLF uses a weighted average computation that is simple to enforce. Secondly, it is a noniterative yet local in nature culminating in much less computational cost when compared to the ones involving iterative architecture [14,43] and global [25] edge-preservation techniques. Thirdly, the fact that BLF can maintain narrow edges while removing noise in the image has been validated. BLF has been applied to a wide range of tasks, including Image Enhancement [44], Artistic Rendering [45], Image Editing [46], Optical Flow Estimate [47,48], Feature Recognition [49], Medical Image Denoising [50,51], and 3D Optical Coherence Tomography Retinal Layer Segmentation [52].

Despite BLF’s strengths, when the job relates to the denoising of the retinal image, BLF may be degraded because it does not take into account the retinal vessel’s special tube-like structure. In most image denoising situations, BLF appears to retain crisp edges when there are two unique features: a prominent contrast found in the vicinity of the border and a bigger region occupied by the edge structure compared to isolated noise. On the other hand, the thin vessels in the retinal image differ from the prevalent crisp edge owing to their weak contrast to the background and the tiny area in the image. BLF could not manage these unique characteristics of the vessels and therefore the information of the vessels would likely have been missed in the blurred picture.

For the detection of vascular structure, various techniques have been investigated. Among these Matched Filter (MF) uses the Gaussian-shaped cross-section of the vessel [53,54,55], detection of reidges based on ridge shaped architecture of the centerlines of vessels [56,57], feature extraction and classification using machine learning algorithms [58,59], and measures of vessels for recognizing tubular vascular structures (via eigenvalues of the Hessian matrix) [60,61]. For instance, MF [33] and Frangi’s Filter (FR) [61] that are among the most common techniques of vessel detection where MF is an efficient method known for its straight forward architecture to detect retinal vessels using a Gaussian-like kernel. Here, the distribution of intensity across the image is mathematically described using an oriented Line Spread Feature (LSF) [62,63,64]. On the other hand, FR is more suited to the detection of of tube-like patterns from the rest of the image parts. That is performed by estimating the unique Hessian matrix values measured for each pixel in the image.

Papillary microvascular deviations caused by Central Retinal Vein Occlusions (CRVO) are analyzed in [65]. Before and after intravitreal Ranibizumab (IVR) injections, vessel density is a measure to investigate the microvascular changes. Segmentation of retinal images has been used in [66] for the diabetic and hypertensive retinopathy. Experiments on DRIVE, CHASE_DB1, and STARE datasets were performed to assess and evaluate the proposed scheme. Similarly, Optical coherence tomography angiography (OCTA) was used by [67] to analyze the various vascular patterns in Retinitis Pigmentosa (RP). Experiments were performed on the high-resolution images obtained from different patients. The proposed scheme was aimed to identify RP vascular anomalies. Velocity and flow of retina are measured using adaptive optics scanning laser ophthalmoscopy (AOSLO) proposed in [68]. In conclusion, the velocity of retinal blood was higher in patients with diabetes (DM). Retina vascular alterations in type 1 Mellitus (T1DM) were investigated in [69]. All patients included in experiments underwent coherence tomography (OCT), microperimetry, dynamic vessel analyzer (DVA), and OCT-angiography (OCT-A). It was concluded that vascular alterations are the core parameters to detect a retinal deviation in DR.

## 3. Vessel Detectors

### 3.1. Improved Frangi Filter

Vessel enhancement filters are defined as scalar functions V:R→R . Their job is to amplify the vessel structure while suppressing any other structure in an image. The filter identifies the vessel structure by examining the 2nd order intensity derivative or Hessian around a given pixel.

Let I(x,y) represent the intensity of a 2-dimensional vessel image, then the Hessian of I(x,y) at scale *s* is defined as:

(1) $$H=\left[\begin{array}{cc} I_{xx} & I_{xy}\\ I_{xy} & I_{yy} \end{array}\right]$$

where $I_{xx},I_{xy}=I_{yx}$ , and $I_{yy}$ all are the second-order derivatives computed on a patch around the pixel of interest. The eigenvalue decomposition of the Hessian matrix provides information about the presence of a vessel structure at the point of investigation. Frangi [61] used the eigenvalues of a Hessian to devise an enhancement filter as provided below.

The most commonly used is the Frangi Filter, which is designed to improve vessels (elongated structures). In this paper, we removed the factor that was implemented to suppress spherical structure and get:

(2) $$V_{F}=\left(1-\exp\left(-\frac{{ℜ}_{A}^{2}}{2\alpha^{2}}\right)\right)\left(1-\exp\left(-\frac{S_{A}^{2}}{2\kappa^{2}}\right)\right)$$

where $S=\sqrt{\lambda_{1}^{2}+\lambda_{2}^{2}+\lambda_{3}^{2}}$ is the 2nd order measure of structure, and ${ℜ}_{A}=\lambda_{2}/\lambda_{1}$ distinguishes between tabular and planar structure. Parameters κ and α control the sensitivity of measure *S* and ${ℜ}_{A}$ .

The Frangi expression is proportional to the strength *S*, which is made up of squared magnitudes of eigenvalues. Thus, it poses a problem for low-contrast vessels, as they are dismissed as noise. Moreover, most vessels have a Gaussian cross-section, that is, they have peak intensity at the center, which then fades gradually as we move towards the edges of the vessel.

In order to remove the dependence of vessel filter on the magnitude of eigenvalues, and at the same time providing better discrimination among vessel and non-vessel class, an improvement is proposed in [61]. In the remainder of the paper $\lambda_{k}$ will be the eigenvalue with the k-th smallest magnitude. The improved Frangi filter is defined as:

(3) $$V=\lambda_{2}^{2}\lambda_{\rho }{\left[\frac{3}{2\lambda_{2}+\lambda_{\rho }}\right]}^{3}$$

If the magnitude of $\lambda_{2}$ and $\lambda_{3}$ is low then the response of such filter is ill-defined and is susceptible to noise in the uniform intensity regions. To overcome this problem the value of $\lambda_{3}$ at each scale *s* is used as:

(4) $$\lambda_{\rho }=\left\{\begin{array}{c} \lambda_{3}\;\;\;\;\;\;\;\;\;\;\;\;\;\;\;\;\;\;\;\;\;\;\;\;\;\;\;\;\;\;\;\;\;\;if\;\lambda_{3}<\tau \;\;\min_{x}\lambda_{x}\left(X,s\right),\\ \tau \;\;\min_{x}\lambda_{x}\left(X,s\right)\;\;\;\;\;otherwise, \end{array}\right.$$

where τ is a cutoff threshold between [0,1] . As, in the case of vessels, the aim is to enhance only bright structures on dark background, having negative eigenvalues, therefore $\lambda_{3}$ with highest magnitude is obtained as $\min_{x}\lambda_{x}\left(X,s\right)$ .

The improved Frangi filter is found exceptionally useful for enhancing low-contrast tiny vessels, as it is based on a ratio of eigenvalues rather than on their magnitude.

### 3.2. Multiscale Line Detector

For the intent of detection, vessels are approximated with a geometric shape called ridges (thin lines darker and brighter than their neighborhood). The best way to detect ridges and remove all other structures is to measure the major eigenvalue of each pixel. The major eigenvalue is a second-order derivative that is oriented in a specific direction, which needs to be pre-smoothed with a Gaussian anisotropic function to boost noise tolerance. This structure results in an elongated Gaussian second-order detector. The filter operates on three parameters: orientation, length σu and width σv . To preserve elongation, the distance of σu is required to be multiples of the width of σv , with amounts of 0.5,1,1.5,2,2.5,3,3.5 . The width parameter σv is chosen from the set 4, 5. The maximum response is chosen for length, width and orientation among all possible sets of values. The generalized two-dimensional Gaussian function is used, provided as follows:

(5) $$g\left(u,v\right)=\frac{1}{2\pi \sigma_{u}\sigma_{v}}\exp^{\left(-\left(\frac{u^{2}}{2\sigma_{u}^{2}}+\frac{v^{2}}{2\sigma_{v}^{2}}\right)\right)}.$$

This generalized Gaussian function poses two independent parameters $\sigma_{u}$ and $\sigma_{v}$ . If we take second derivative with respect to *u* only, the following expression is obtained.

(6) $$g_{uu}\left(u,v\right)=\frac{1}{2\pi \sigma_{u}^{5}\sigma_{v}}\left(u^{2}-\sigma_{u}^{2}\right)\exp^{-\left(\frac{u^{2}}{2\sigma_{u}^{2}}+\frac{v^{2}}{2\sigma_{v}^{2}}\right)}.$$

The discrete kernel is rotated as u=xcosθ−ysinθ and v=xsinθ+ycosθ in a particular direction. The output α and β are calculated for ideal ridge patterns in [70], and the values are α=1.5 and α=0.5 . Nonetheless, our emphasis here is to increase the intensity of the detector for small-width vessels that also have a lower contrast. To this end, for our database images α=1 and β=0.5 seems more appropriate. Using these scale-normalization parameters, the maximum response is calculated for each pixel, analyzing the different combinations of size, width, and orientation.

## 4. Theory and Methodology

In this work, we propose an enhanced framework for computerized identification of eye diseases from retinal fundus images by employing S-BM3D denoiser in combination with unsupervised methods for detecting retinal vessels. Previously, researchers and medical scientists have developed automated methods whereby pipelines of specialized processes are used on the fundus images for detecting and segmenting retinal vessels. This leads to the prediction about the presence of pathologies within an acceptable degree of precision. The challenge in this regard is the presence of noise and poor contrast regions within the fundus images. Specifically, retinal fundus images are known to be corrupted by the multiplicative speckle noise during their acquisition whereby the traces of systematic additive noise are also observed [31,32,71]. Consequently, the noise of varying nature with high magnitude prohibits the computerized detectors from identifying the tiny vessels, which limits their widespread use. To remedy that, we propose the use of the gold standard S-BM3D filter on fundus images owing to its ability to recover image details (i.e., vessels). In doing so, we address the inherent checkerboard artifact problem in S-BM3D by suggesting a novel ensemble filtering approach to mitigate these artifacts without compromising the quality of the recovered details. Naturally, we first describe the S-BM3D architecture followed by the formulation of the proposed ensemble S-BM3D filter and the enhanced unsupervised vessel segmentation framework.

### 4.1. Speckle Adapted Block Matching 3D (S-BM3D) Denoiser

The *S-BM3D* filter, named in the sequel, follows the multi-step procedure introduced in the original BM3D filter [29] that was designed to cater for additive white Gaussian noise (wGn). Owing to its superior performance, this multi-step complex procedure that involves block matching and 3D collaborative (BM3D) filtering, has become a gold standard in image denoising. Naturally, it has seen many variants addressing the cases of non-Gaussian noises encountered in practice. Among those, the *S-BM3D* filter [30] is developed for restoring the speckled synthetic aperture radar (SAR) images. This method seeks to exploit the efficacy of the BM3D method [29] by theoretically incorporating the statistics of speckle within its superior architecture. Consequently, the *S-BM3D* method is among the top despeckling methods, which is precisely the rationale behind its use in this research. To impart a better understanding of how the *S-BM3D* filter removes the noise from the fundus image as part of the proposed research, each step is discussed as a separate subsection as follows.

#### 4.1.1. Block Matching

Natural as well as medical images are largely composed of redundant or similar regions spread across the image. This property of an image is generally characterized as self-similarity, i.e., repetition of most regions over and over again across the image. The *S-BM3D* filter takes advantage of this representation by identifying the similar blocks through a distance measure. Next, similar regions are processed simultaneously as a group for noise removal. Specifically, similar patches are rearranged as a single 3D patch for it to be processed by a collaborative 3D filter.

To identify the similar patches, the speckle version of the *BM3D* departs from the notion of minimum Euclidean distance between two patches. That is well adapted to the AWGN case but is not suitable for speckle noise. Contrarily, the *S-BM3D* filter employs a probabilistic approach inspired by [26]. Thereby, the probability distribution of the amplitude $a\left(\cdot \right)=\sqrt{z(\cdot )}$ , such that $z\left(\cdot \right)$ is a speckled or noisy pixel, is modeled using the square root Gamma distribution with order *L*. Afterward, the likelihood that two pixels from two different observations (patches centered at locations *s* and *t* in space) belong to the same noiseless background that is used to derive the probabilistic distance *D*, as given in (7).

(7) $$\begin{array}{cc} D=\sum_{k} & [\left(2L-1\right)\log\left(\frac{a\left(s+k\right)}{a\left(t+k\right)}+\frac{a\left(t+k\right)}{a\left(s+k\right)}\right)\\ & +\gamma L\left(\frac{\hat{x}\left(s+k\right)-\hat{x}\left(t+k\right)}{\hat{x}\left(s+k\right)\hat{x}\left(t+k\right)}\right)] \end{array}$$

where γ weighs the importance of data over the prior and z(k)=x(k).u(k) such that x(k) denotes the clean pixels and u(k) denotes the speckle noise at spatial location *k*.

#### 4.1.2. Collaborative Wavelet Shrinkage

This step does not perform the noise removal, instead it is carried out to merely obtain an estimate of the clean image. This is required as a prior in the subsequent step within the Weiner filter based noise removal. To obtain an estimate of the true image, the *BM3D* method performed hard thresholding in the wavelet domain, which is a suitable approach in the case of AWGN removal. However, in the case of multiplicative speckle noise, hard thresholding is not properly motivated. Consequently, the *S-BM3D* filter employs the local linear minimum mean squared error (LLMMSE) estimator for shrinkage of wavelet coefficients, which is well adapted to speckle removal. Furthermore, the wavelet decomposition in this step was performed using the redundant wavelet transform to obtain a more reliable estimate of the clean image as a prior for the next step.

#### 4.1.3. Collaborative Wiener Filtering

This collaborative filtering step also has an LLMMSE shrinkage form but with a prior estimate of the true or noiseless image estimated in the previous step. As a consequence of the availability of the estimate of the true coefficients, the LLMMSE shrinkage function amounts to an empirical Weiner filter in the wavelet domain. Following, the collaborative filter is applied on the *i*th 3D block Z(i) to obtain the final noiseless estimate $\hat{X}\left(i\right),$

(8) $$\hat{X}\left(i\right)=\frac{{\hat{X}}^{\prime^{2}}\left(i\right)}{{\hat{X}}^{\prime^{2}}\left(i\right)+\langle V^{2}\rangle }\;Z\left(i\right),$$

where ${{\hat{X}}^{\prime }}^{2}\left(i\right)$ is the prior noiseless estimate obtained in the previous step while $\langle V^{2}\rangle$ is the expectation of the squared difference between the prior ${{\hat{X}}^{\prime }}^{2}\left(i\right)$ and noisy coefficient $Z\left(i\right)$ .

#### 4.1.4. Aggregation

This type of collaborative filtering, discussed in the previous step, may lead to the spread of a pixel value to more than one block owing to its estimations as a part of multiple blocks. Therefore, in this step, these estimates of a pixel are averaged with appropriate weights to obtain the denoised pixels, as follows

(9) $$\hat{x}\left(k\right)=\frac{1}{N}\sum_{m\in M(k)}w_{m}{\hat{x}}_{m}\left(k\right)$$

where ${\hat{x}}_{m}\left(k\right)$ is the estimate of clean pixel x(k) from the block indexed by m∈M(k) where M(k) is the group of *N* blocks containing the noisy version of x(k) (i.e., z(k) ).

**Figure 1:**
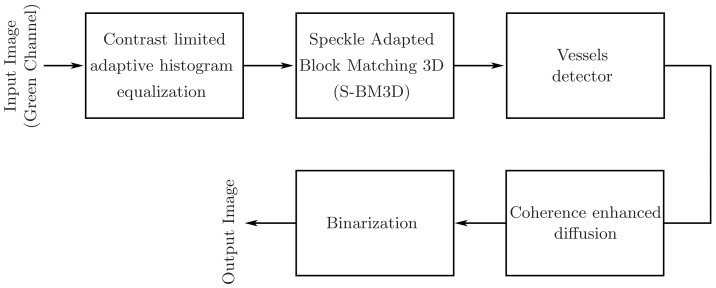
Block diagram of the proposed retinal vessel segmentation strategy.

This sophisticated and cultivated mechanism used within the *BM3D*-speckle filter not only minimizes the noise but also successfully preserves smaller vessels previously obscured by noise. As a consequence, the uncovered smaller vessels are now exposed to detection and segmentation, which may result in enhanced accuracy of the computerized eye disease detectors. However, the *S-BM3D* filter results in an abundance of checkerboard artifacts throughout the denoised image that presents a challenge in the use of the *S-BM3D* method on fundus images for the improvement of the segmentation of blood vessels. Specifically, these artifacts deteriorate the already diminished edges of fine vessels and, as a consequence, may lead to either loss or false detection of tiny vessels during segmentation. Hence, doing away with these artifacts is necessary to use the *BM3D*-speckle filter as part of the suggested pipeline in Figure 1 for an improved disease detection performance.

### 4.2. An Ensemble Bock-Matching 3D Speckle (ES-BM3D) Filter for Fundus Images

Fundus images are severely affected by speckle noise due to scattering of the reflected light that distorts or conceals smaller vessels. As a consequence, these smaller vessels are not detected during segmentation. To address this issue we suggest the use of the state-of-the-art S-BM3D filter that can minimize speckle without compromising on the finer details (i.e., vessels in this case). However, the cost of recovering fine vessels, owing to its complicated multi-stage processing, are the checkerboard artifacts. These high density artifacts discourage the use of the S-BM3D filter in sensitive applications involving vessel segmentation and eye disease detection from retinal fundus images. Hence, in this work, we propose to alleviate this inherent issue within the S-BM3D filter via a customized ensemble averaging filter [35]. To describe the proposed approach we begin by discussing the motivation behind the use of S-BM3D denoiser on fundus images and the challenges that lead to the development of the proposed ensemble S-BM3D (ES-BM3D) approach for fundus image denoising.

#### 4.2.1. Rationale

We employ the S-BM3D filter for fundus image denoising owing to its ability to preserve the finest details while doing away with multiplicative speckle noise. That is why S-BM3D has been regarded as the gold standard speckle denoiser since its advent. In comparison, the PPB denoiser that was used in [34] for improved vessel segmentation, over-smooths the image details leading to the loss of finer details. Similarly, many other state-of-the-art despeckling methods suffer from the same limitation of losing finer details [15,19,28]. However, the robust architecture within the S-BM3D filter ensures retention of fine details with the maximal removal of speckle noise. The catch here, though, is a pattern of widespread artifacts due to S-BM3D that presents a serious challenge in detecting smaller vessels. On the contrary, these artifacts are ignored in the case of SAR images owing to their less sensitive applications.

**Figure 2:**
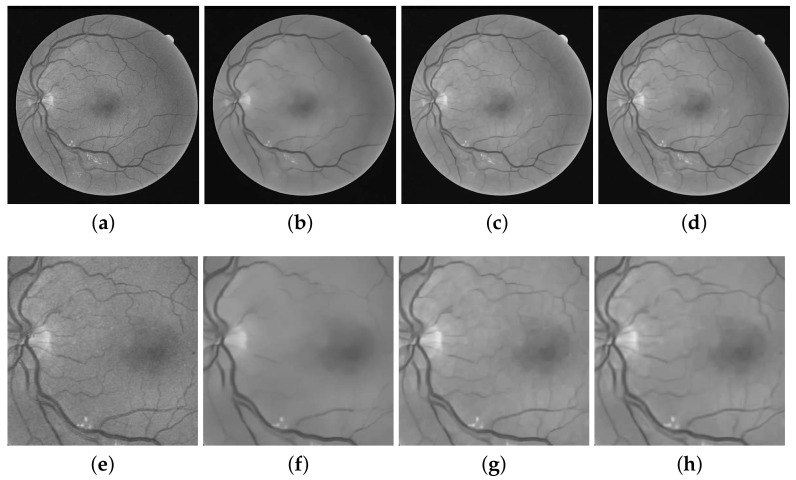
A toy example to motivate our work where the top row shows actual noisy and denoised images from various methods while the bottom row gives zoomed views of the selected region from the images in the top row. First, we show the deterioration caused by noise (in sub-figures **a**,**e**), which show a noisy fundus image from the DRIVE dataset. Subsequently, we show how well the denoised images from the S-BM3D (shown in subfigures **c**,**g**) retain tiny vessels when compared to the PPB denoiser that loses most of the tiny vessels (see sub-figures **b**,**f**). Finally, we shift your attention to the checkerboard artifacts through the zoomed-in views (in the bottom row) in the results of the S-BM3D method and the distortion caused by these artifacts. This motivates our proposed ensemble S-BM3D filter (the results of which are shown in sub-figures **d**,**h**), which successfully suppresses these artifacts while retaining all the vessels.

To give more insight into the matter, we present a toy example in Figure 2 where a fundus image from the DRIVE database is shown along with its denoised versions by various methods. The sub-figures shown in the lower row of Figure 2 are the zoomed in view of the specific region of the corresponding image in the top row. This example is specifically presented to show how well the S-BM3D recovers each vessel from the noisy fundus image while suppressing the noise. Moreover, this toy example also highlights the problem of checkerboard-like-artifacts and its impact on vessel segmentation. Consequently, we propose an ensemble framework to minimize these artifacts due to the S-BM3D framework. We next discuss the results in Figure 2 to demonstrate the extent of artifact problem that will serve as a motivation of the proposed approach.

Observe that noisy image in Figure 2a shows significant distortion due to noise that eludes the detection of tiny vessels. Evidently, from Figure 2c,g, the S-BM3D filter recovers almost all of the finer vessels while successfully eliminating noise. On the contrary, the PPB denoiser records the loss of majority of the finer vessels while doing away with speckle noise, see Figure 2b,f. However, the challenge in the S-BM3D filter is the checkerboard-like-artifacts, which are apparent from Figure 2c (and its zoomed-in view in Figure 2g). These artifacts may be seen as the cost paid for such a complicated mechanism to recover tiny image details. Thus, posing a serious challenge in detection of fine vessels. That is because these artifacts deteriorate edges or end points of fine vessels, which can be observed from the zoomed-in views in Figure 2g. This negatively impacts the detection of these vessels, i.e., false detection or rejection of vessels. Hence, in order to employ the superior framework of the S-BM3D filter for vessel segmentation, these artifacts must be alleviated.

#### 4.2.2. Customized Ensemble Filter

To address this issue of checkerboard artifacts, we propose a customized ensemble filtering approach that suppresses checkerboard artifacts observed within the S-BM3D denoised image. The idea of ensemble filtering is inspired by the cycle spinning operation in [35], which is used to suppress the artifacts due to the lack of translation invariance of the wavelet decomposition. In cycle spinning operation, the noisy image/signal is shifted to obtain its various copies, each of which is denoised leading to their ensemble averaging to get an improved image with minimum artifacts.

**Figure 3:**
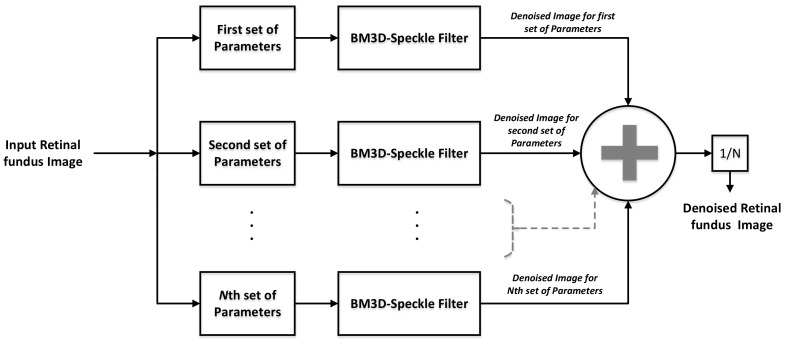
Block diagram of the proposed ensemble-block matching 3D (ES-BM3D) method that employs an ensemble of the speckle adapted (S)-BM3D filter to minimize noise.

The proposed ensemble filter is built on the same lines where ensemble averaging is used as a means to suppress artifacts. Although, we do not employ the shift and the average operation used in [36,37] because it essentially works as a low pass filter that removes finer image details along with the artifacts. The proposed approach, on the contrary, devises a customized ensemble averaging framework that intelligently removes artifacts without recording much loss in the recovered image details. In this regard, multiple denoised versions of the noisy fundus image are obtained using a combination of S-BM3D filters, each of which are tuned at different parameters. The block diagram of the proposed ensemble approach is shown in Figure 3 where it is shown that a fundus image is denoised by the S-BM3D filter for a variety of parameters that culminates on averaging of all the denoised versions.

In this regard, the parameters controlling the trade-off between over-smoothing (i.e., loss of finer vessels) and retention of finer details are varied in each iteration as represented by the parameter block in Figure 3. Specifically, this is triggered by varying the block size and search window parameters of the S-BM3D filter. This results in a few denoised images containing finer details along with the artifacts while others are oversmoothed versions with fewer artifacts. Now ensemble averaging these denoised versions results in alleviation of the problematic artifacts without compromising on the finer edges or vessels.

To demonstrate how well the ensemble filter suppresses the artifacts, while retaining the vessels within the fundus image, we show the denoised image from the proposed ES-BM3D filter and its zoomed-in view in Figure 2d,h. Observe that the ES-BM3D filter significantly mitigates the artifacts shown within the S-BM3D results in the S-BM3D filter, respectively, in Figure 2c,g. The zoomed-in view further highlights the facts that the edges of the tiny vessels are not affected by these vessels, see Figure 2h. Next, we use the proposed approach within an unsupervised vessel segmentation strategy to improve its performance.

### 4.3. Improved Retinal Vessel Segmentation Strategy Based-on the Proposed ES-BM3D Denoiser

The complete architecture illustrating various processing units that starts the flow process with an input color fundus image and ends with a binary vessel image is depicted in Figure 3. In the first step, the contract of the input image is enhanced. Improving image contrast is a vital preprocessing step that is widely used in pattern recognition, medical imaging, and computer vision. The aim is to enhance the overall appearance of the image without any over or under-enhancement, while at the same time keeping the noise gain to a minimum. Weak image sensors, uneven exposure, and poor ambient light are a few of the many factors contributing to a distorted contrast image and poor dynamic range. CLAHE is an enhanced type of adaptive histogram equalization (AHE) developed by K. Zuiderveld [72] to enhance low contrast biomedical images. By dividing the image into small interrelated areas called tiles, and then applying histogram equalization across each tile, CLAHE reduces the noise amplification issue. In the pre-processing step, CLAHE was chosen by many researchers as it produces more prominent hidden features and edges by enhancing local contrast and making full use of the available grey level spectrum. After enhancing the contrast, the image is denoised by Speckle Adapted Block-Matching. The denoised image is then passed to a vessel detector. For vessel detection, we used two detectors, a multiscale line detector and a modified Frangi detector. Anisotropic diffusion has been used to make the vessel’s intensity uniform across its length. Anisotropic diffusion is a technique designed to reduce noise within the vessel without removing significant parts of the vessel. In a general sense, the anisotropic filter is more like a locally adapted filter that adopts anisotropic behavior close to linear structures such as vessels, i.e., its support region is elongated along the vessel and narrow across the vessel, preserving the general shape of the vessels while smoothing the noise within the vessel area.

After dissection the final step is finalization. An iterative method for threshold selection has been proposed by Ridler and Calvard in [73] for object-background discrimination. The image contains intensity values in the range [0,L] . The distribution of gray-levels is given by the histogram *h*, where h(0),h(1),⋯,h(L) are the histogram points with gray levels 0,1,⋯,L . Let [LO,UP] be the smallest interval containing all non-zero histogram values. The ISODATA algorithm can then be described as:

- Choose some initial value for the mean μ such that LO≤μ≤UP . In this research work, we choose the Otsu method to provide us with the initial value for the mean.
- Calculate threshold *T* by the formula:
(10) $$T=\frac{\mu_{0}+\mu_{1}}{2}.$$
  Based on threshold *T*, the image in FOV has been divided into vessel and non-vessel regions.
- Two new mean values were computed, $\mu_{0}$ and $\mu_{1}$ based on the regions formed by threshold *T*.
- If any of the mean values are changed, go to loop; otherwise stop.

The initial value for the threshold $T_{0}$ is chosen by selecting a region that is mostly likely to contain pixels of only background class.

## 5. Results and Discussion

### 5.1. Denoising Results

For experimental validation, we compare the performance of the proposed ES-BM3D denoiser with state-of-the-art speckle denoisers including the PPB and the S-BM3D. The input images are taken from the DRIONS dataset of noisy fundus images [74]. Owing the unavailability of the ground truth or noiseless image, the quantitative performance analysis in terms of peak signal to noise ratio (PSNR), structural similarity (SSIM) index, etc., cannot be performed. Hence, we demonstrate the denoising results by visually displaying the denoised images by various methods along with the noisy fundus image. That way, the quality of the denoised image from a comparative method can be observed visually.

**Figure 4:**
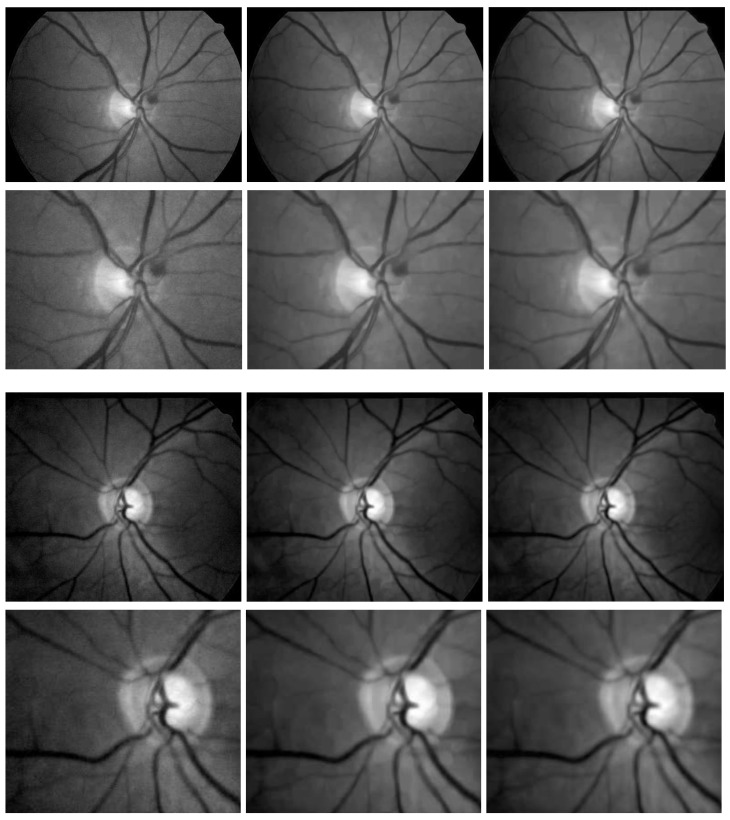
Comparison of visual denoising results of the proposed ES-BM3D (shown in the second column) against the S-BM3D method (shown in the third column) on two noisy (fundus) images (shown in first column) from the DRIONS dataset [74]. The figure is arranged such that the first and third rows show the noisy and denoised images while the second and third row shows the zoomed in views of specific regions from the images in their top rows.

Firstly, we compare the performance of the proposed method with the S-BM3D denoiser in Figure 4 whereby the first column displays two different noisy fundus images (in first and third row) while the second and third columns display corresponding denoised images from the S-BM3D denoiser and ES-BM3D denoiser (in first and third row). For a deep insight, we also give zoomed-in views of the noisy and denoised images in the second and fourth (bottom) rows. Observe that the denoised images from the S-BM3D filter preserves almost all the details (i.e., vessels) but at the cost of visible checkerboard artifacts, see images and their zoomed-in views in the second column of Figure 4. The denoised images from the proposed ES-BM3D denoiser and their zoomed-in views, in the third column of Figure 4, significantly minimizes the checkerboard patterns observed in sub-figures in the second column. At the same time, the proposed ES-BM3D denoiser manages to retain all the image details (i.e., vessels) owing to the intelligently customized ensemble filter, which is a major contribution of our work.

**Figure 5:**
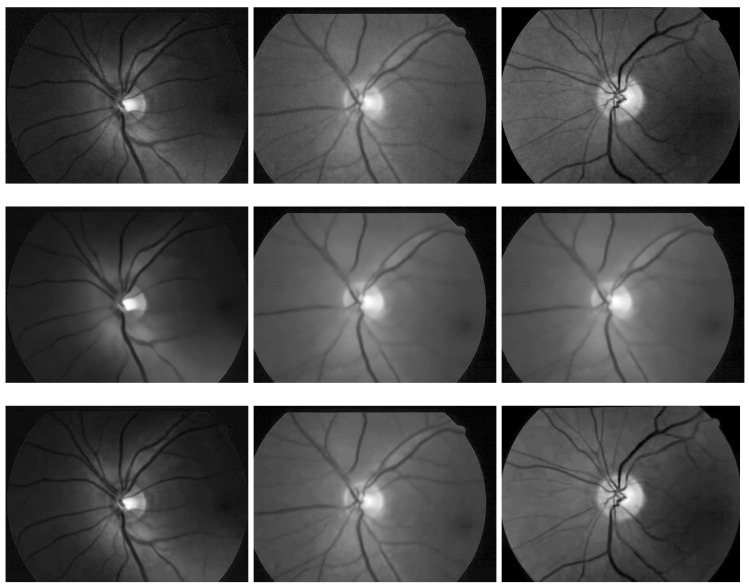
Comparison of the denoising performance of the proposed ES-BM3D against the probabilistic patch based (PPB) method on a few noisy fundus images from the DRIONS dataset [74]. The figure is arranged such that the noisy images are placed in the top row; the denoised versions by the PPB method are shown in the middle row while the denoised images by the proposed ES-BM3D are placed in the last row.

Next, we compare the proposed ES-BM3D approach against the PPB filter (used for vessel segmentation in [34]) in Figure 5. Thereby, the first row shows noisy fundus images, the second row shows the corresponding denoised images from the PPB denoiser and the third (bottom) row displays denoised images from the proposed ES-BM3D filter. It is clear from the figure that the PPB denoiser removes noise at the cost of the majority of the finer vessels. The denoised images in the second column of Figure 5 have lost the smaller vessels while retaining the larger vessels, which explains its use in [34] for detecting large vessels. On the contrary, the proposed approach suppresses noise without any significant loss of vessels, which can be observed from the third column of Figure 5 where noise is completely removed and majority of vessels (including smaller ones) are still intact. In addition, these images do not depict any signs of artifacts earlier observed in the results of the S-BM3D filter.

### 5.2. Retinal Vessel Segmentation Results

#### Materials

The proposed methodology has been tested on different groups of image samples that are publicly available and described as follows.

1. **STARE (Structured Analysis of Retinal)** [75]: For the purpose of sampling, 20 mid-resolution images were extracted from a set of 400 images collected in USA.
2. **DRIVE (Digital Retinal Images for Vessel Extraction)** [76]: Periphery scans of the retina were extracted from group diabetics collected as broad age group diabetics in Netherland.
3. **CHASE** [77]: 28 sample images were taken from the CHASE dataset originally provided by the Kingston University, London.

In DRIVE, each image is accompanied by binary masks. The vascular structure is manually segmented into a vessel and non-vessel oriented boolean image in each available image demarcating the Field of View (FOV) zone. Contrasting DRIVE, the boolean FOV mask is unavailable for the STARE database. Consequently, its corresponding mask must be generated employing existing procedures [58]. It is noteworthy that the trial input patch may be originated from any region of the image and unconfined within their masks. The system is geared towards learning so as to evolve an efficient discriminating algorithm among the edges of the mask and blood vessels in the retinal images.

### 5.3. Evaluation Criterion

The performance of any vascular segmentation technique relies on its ability to correctly distinguish between vessels and background pixels. The performance measures are compared with the manually annotated ground truth binary masks which act as reference maps. This distinction results in the core values of true/false and positive/negative. A pixel marked as a vessel is labelled positive, while identification as a background pixel is labelled as a false category. True means right segmentation of any pixel as either a vessel or a non-vessel, and vice versa. Thus, all four variations of these variables play an important role in assessing the effectiveness of any technique of vascular classification:

1. True Positive (TP): when Vessel is correctly classified,
2. False Negative (FN): when vessel is classified as background,
3. True Negative (TN): when Non-vessel is correctly classified,
4. False Positive (FP): when Non-vessel is classified as vessel.

With the above mentioned core parameters, precise ratios are assessed in order to measure and compare the efficiency of the examined technique with other state-of-the-art segmentation strategies as [77]:

$$\begin{array}{c} Se=\frac{TP}{TP+FN},\\ Sp=\frac{TN}{TN+FP},\\ Acc=\frac{TP+TN}{TP+FN+TN+FP}, \end{array}$$

High sensitivity value (or TPR) implies better vessel segmentation capability, and the same applies to precision (or 1-FPR) in terms of classifying background pixels. The ratio of all pixels correctly categorised as vessels or backgrounds and the total pixels in the field of view (FOV) give the algorithm precision.

### 5.4. Comparison with State-of-the-Art

**Figure 6:**
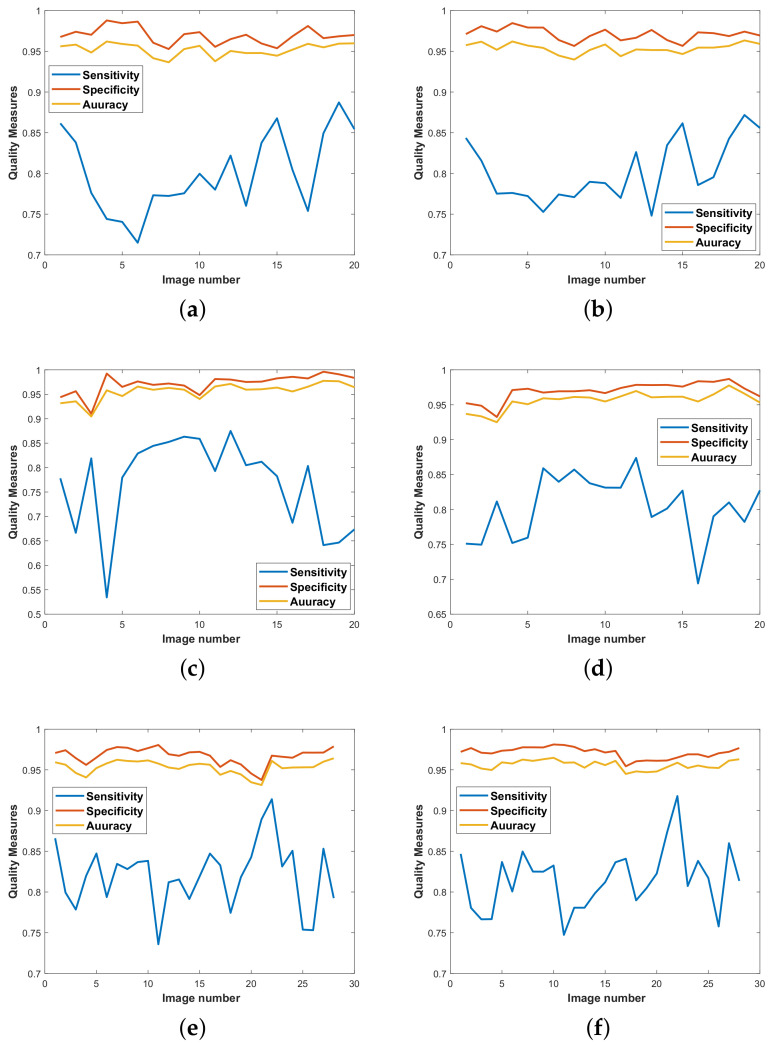
Quality measures for datasets: (**a**) modified Frangi on DRIVE, (**b**) multiscale line detector on DRIVE, (**c**) modified Frangi on STARE, (**d**) multiscale line detector on STARE, (**e**) modified Frangi on CHASE, (**f**) multiscale line detector on CHASE.

To evaluate the performance of the proposed framework, the system has been tested using DRIVE, STARE and CHASE_DB1 databases. Quality measures for all three mentioned datasets have been presented in Figure 6. To authenticate the performance, statistical parameters; sensitivity (Se), specificity (Sp), accuracy (Acc) and area under curve (AUC) are considered. Experimental results on the given datasets are summarized in Table 1. Moreover, the results have been compared with state-of-the-art vascular segmentation algorithms, which can be seen in Table 2, Table 3 and Table 4. In Table 2, Table 3 and Table 4, the highest three results are shown by three color codes (red, green, blue) where red color represents first highest result, green second high and blue third highest value.

It can be seen in Table 2, Table 3 and Table 4, the proposed algorithm generally produced efficient results using all three benchmark databases than the rest of the unsupervised algorithms. The sensitivity (Se) values generated by the proposed method for STARE, CHASE_DB1 and DRIVE database are 0.8084, 0.8012 and 0.8007, respectively. These results show that the proposed system outperforms all the unsupervised algorithms on DRIVE and CHASE_DB1 datasets, and only on the STARE dataset, it is almost near the top result of [90]. The specificity (Sp) score generated by the proposed method is about 0.9778, 0.9730 and 0.9721 respectively. The specificity (Sp) results placed the proposed method on second and third top places among other state-of-the-art methods using CHASE_DB1 and STARE datasets.

As to Accuracy (Acc) of the proposed method, the scores stand around 0.9600, 0.9578 and 0.9571, respectively. The Acc (0.9600) of the proposed method follows [87], which has an Acc value of about 0.97 using the DRIVE database. At the same time, the Acc values 0.9578 and 0.9571 placed the proposed method on the third spot in Table 3 and Table 4 using CHASE_DB1 and STARE databases. On the other hand, for the same datasets, the highest accuracy has been generated by [87,90] with Acc values 0.97 and 0.9691, respectively. The average time required to segment an image on a PC (Intel Core i7, 2.21 GHz with 16 GB RAM) is approximately 2.70 s. That compares well with computationally expensive state of the art methods in Table 5. These methods were implemented using MATLAB2017a.

## 6. Discussion

In this paper, a novel approach has been proposed to detect retinal vessels using unsupervised learning methods. Usually, some pixels are lost when detecting retinal vessels because of noise and anomalies in retinal vessels during pathologies. The proposed scheme is focused on retinal vessels to correctly detect the edges so that the detection rate of retinal vessels and background pixels is improved. Moreover, this work aims to present a framework that improves the performance of classical detectors. In this work, we demonstrate the efficacy of our method with FRANGI and MULTISCALE methods but our framework is equally applicable to more evolved vessel recent detectors (i.e., [87,90]) to further improve their performance.

According to Figure 6, the proposed scheme is evaluated on different samples from datasets described in Section 5. A quality measure for both techniques—FRANGI and MULTISCALE—is calculated on each dataset. The maximum value of quality measure for FRANGI and MULTISCALE on the DRIVE dataset is calculated as 0.89 (Figure 6a) and 0.88 (Figure 6b), respectively. The quality measure values for FRANGI and MULTISCALE on the STARE dataset are calculated as 0.88 (Figure 6c) and 0.88 (Figure 6d in order. Similarly, both techniques are evaluated on the CHASE_DB1 dataset and the quality measure for both FRANGI and MULTISCALE is 0.92 (Figure 6e,f).

**Figure 7:**
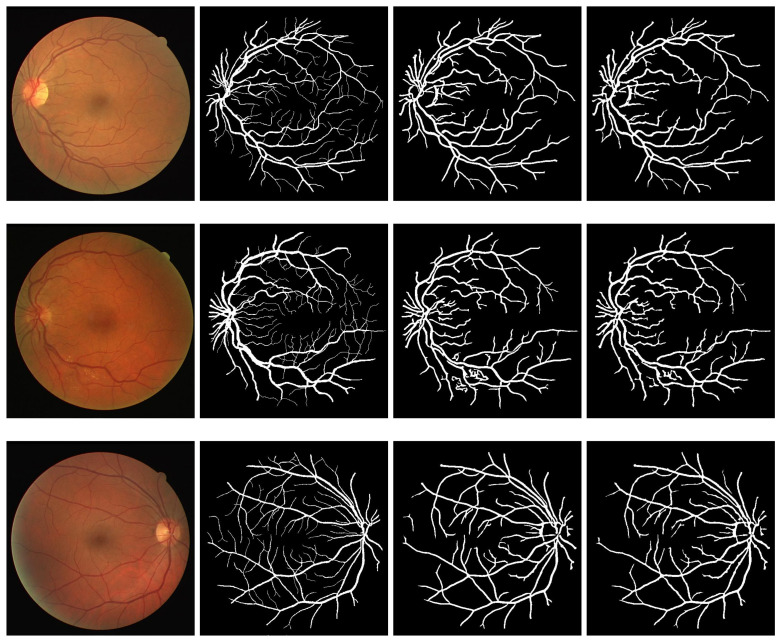
Segmentation results of DRIVE: column 1 shows three sample images, column 2 shows the ground truth images corresponding to column 1, column 3 shows the output result modified Frangi filter and column 4 shows the result of multiscale line detector.

**Figure 8:**
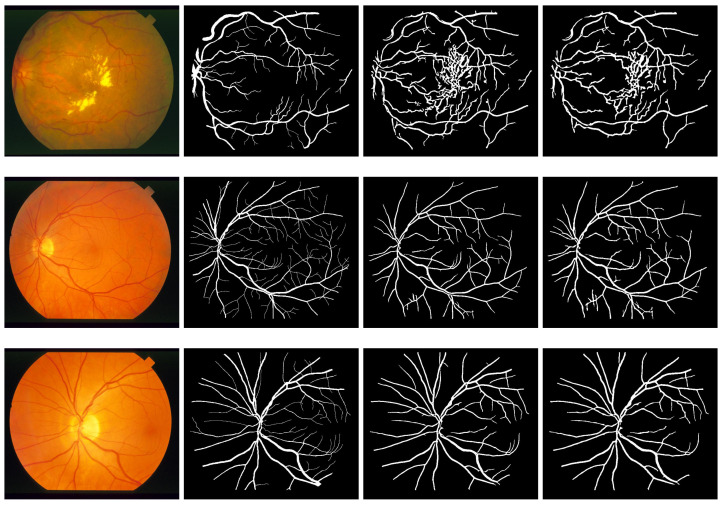
Segmentation results of STARE: column 1 shows three sample images, column 2 shows the ground truth images corresponding to column 1, column 3 shows the output result modified Frangi filter and column 4 shows the result of multiscale line detector.

**Figure 9:**
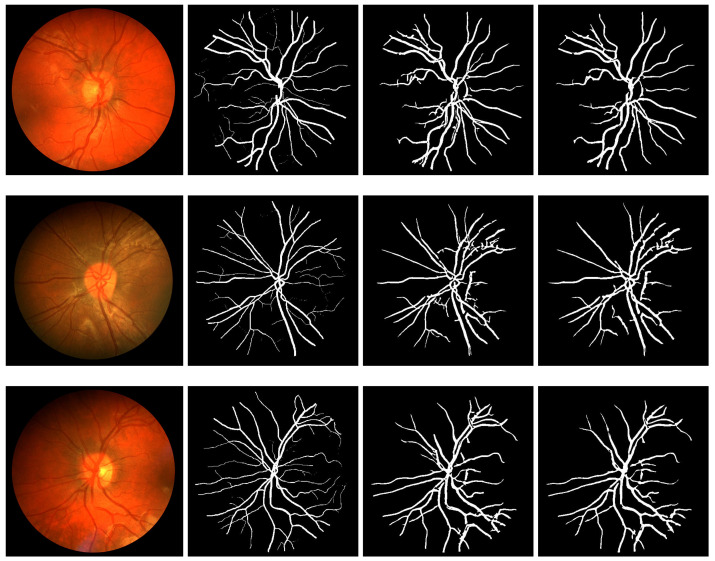
Segmentation results of CHASE: column 1 shows three sample images, column 2 shows the ground truth images corresponding to column 1, column 3 shows the output result modified Frangi filter and column 4 shows the result of multiscale line detector.

Similarly, results obtained from segmentation performed on DRIVE, STARE, and CHASE_DB1 datasets are presented in Figure 7, Figure 8 and Figure 9, respectively. In each of the figures, column 1 shows three sample images, where column 2 shows the ground truth images corresponding to column 1. Column 3 shows the output results of the modified Frangi filter and finally, column 4 depicts the result of a multiscale line detector.

**Figure 10:**
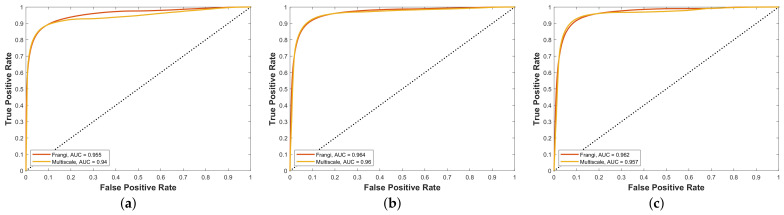
The comparison of the proposed Frangi and multiscale methods in terms of area under the ROC curve on the DRIVE (**a**), STARE (**b**) and CHASE (**c**) datasets, respectively.

In order to support the comprehensive performance of the proposed methods on the unbalanced task of vessel segmentation, we present their reciever’s operating characteristic curves along with area under the curve for all datasets in Figure 10. It is evident that the proposed Frangi and Multiscale methods work well independently on the complete range of the segmentation thresholds.

**Figure 11:**
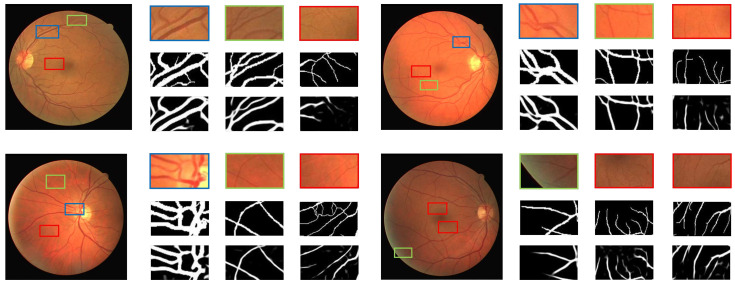
Visual illustration of the segmentation quality of the proposed method on thick, medium and tiny vessels. Blue regions contain thick vessels, green rectangles encapsulate representative examples of vessels with medium thickness and finally thin or tiny vessel are depicted by red bounded boxes. This figure is best viewed in color.

In order to demonstrate the performance of the proposed segmentation method on vessels of different thickness and complex vessel structures, we show the magnified visual results of the proposed method on representative fundus images in Figure 11. For each image the first row presents the cropped regions containing various vessel structures with varying thickness, the second row shows the manual annotation while the third row presents the visual outputs of the proposed method. It can be clearly observed that the proposed method robustly captures thick and medium vessels for all representative images with accurate details. It is noted that some fine-grained details are missed by the proposed method in the case of thin vessels for a few representative images. However, representative images three and four demonstrate that the proposed method is able to preserve thin vessel information boosting its average performance.

## 7. Conclusions

In this paper, we have devised a new strategy by introducing a noise removal methodology using the speckle adapted block-matching 3D (S-BM3D) filter that precedes the vessel segmentation step by mitigating the effect of noise. This step significantly boosts the efficiency of a multiscale line detector as well as Frangi’s vessel detection capabilities. All experiments were performed on three well-established clinical and publicly available datasets; DRIVE, STARE, and CHASE_DB1. Experimental results were evaluated and compared with state-of-the-art methods. We achieved a maximum value of sensitivity of 80.84 and accuracy value of 96. The performance of the proposed scheme was observed with significant enhancement, especially in sensitivity and accuracy. Thus, the evaluation metrics of the proposed method surpassed similar state-of-the-art methods. Moreover, the proposed ensemble filtering framework is fully data driven and does not require tuning of any parameter apriori, which makes it equally applicable to all type of datasets.

## Figures and Tables

**Table 1:** Evaluation matrix.

		ES-BM3D (FRANGI)	ES-BM3D (MULTISCALE)
**STARE**	**Specifity**	0.9649	0.9653
	**Sensitivity**	0.8056	0.8288
	**Accuracy**	0.9526	0.9541
**DRIVE**	**Specifity**	0.9777	0.9702
	**Sensitivity**	0.7687	0.8141
	**Accuracy**	0.9570	0.9540
**CHASE_DB1**	**Specifity**	0.9672	0.9711
	**Sensitivity**	0.8203	0.8153
	**Accuracy**	0.9531	0.9561

**Table 2:** Comparison with the most recent techniques on the DRIVE dataset where highest three results are shown by three color codes (red, green, blue) such that red color represents first highest result, green second high and blue third highest value.

Type	Methods	Year	Se	Sp	Acc	AUC
**Supervised methods**	Li [78]	2016	0.7569	0.9816	0.9527	0.9738
	Orlando FC [79]	2017	0.7893	0.9792	N.A	0.9507
	Orlando UP [79]	2017	0.7076	0.9870	N.A	0.9474
	Dasgupta [80]	2017	0.9691	0.9801	0.9533	0.9744
	Yan [81]	2018	0.7653	0.9818	0.9542	0.9752
	Hu [82]	2018	0.7772	0.9793	0.9533	0.9795
	Oliveira [83]	2018	0.8039	0.9804	0.9576	0.9821
	Alom [84]	2018	0.7792	0.9813	0.9556	0.9784
	Li [85]	2019	0.8349	N.A	0.9563	0.9157
	Jiang [86]	2019	0.7839	0.9890	0.9709	0.9864
**Unsupervised methods**	Zhang [77]	2016	0.7743	0.9725	0.9476	0.9636
	Karn [87]	2018	0.78	**0.98**	**0.97**	0.88
	Aguiree [88]	2018	**0.7854**	0.9662	0.950	N.A
	Khan [89]	2018	0.730	**0.979**	**0.958**	0.855
	Hashemzadeh [90]	2019	**0.7830**	**0.9800**	0.9531	0.9752
	**Proposed (FRANGI)**	2019	0.7687	0.9777	**0.9570**	0.9554
	**Proposed (MULTISCALE)**	2019	**0.8141**	0.9702	0.9540	0.9399

**Table 3:** Comparison with state-of-the-art methods on the CHASE_DB1 dataset. Here, highest three results are shown by three color codes (red, green, blue) such that red color represents first highest result, green second high and blue third highest value.

Type	Methods	Year	Se	Sp	Acc	AUC
**Supervised methods**	[78]	2016	0.7507	0.9793	0.9581	0.9716
	[79] FC	2017	0.7277	0.9712	N.A	N.A
	Alom [84]	2018	0.7756	0.9820	0.9634	0.9815
	[81]	2018	0.7633	0.9809	0.9610	0.9781
	Oliveira [83]	2018	0.7779	0.9864	0.9653	0.9855
	Jiang [86]	2019	0.7839	0.9894	0.9721	0.9866
**Unsupervised methods**	Zhang [77]	2016	0.7626	0.9661	0.9452	0.9606
	Karn [87]	2018	**0.78**	**0.97**	**0.97**	N.A
	Hashemzadeh [90]	2019	0.7737	**0.9840**	**0.9623**	0.9789
	**Proposed (FRANGI)**	2019	**0.8203**	0.9672	0.9531	0.9621
	**Proposed (MUTLISCALE)**	2019	**0.8153**	**0.9711**	**0.9561**	0.9565

**Table 4:** Comparison with state-of-the-art methods on the STARE dataset where highest three results are shown by three color codes (red, green, blue) such that red color represents first highest result, green second high and blue third highest value.

Type	Methods	Year	Se	Sp	Acc	AUC
**Supervised methods**	Li [78]	2016	0.7726	0.9844	0.9628	0.9879
	Orlando FC [79]	2017	0.7680	0.9738	N.A	N.A
	Orlando UP [79]	2017	0.7692	0.9675	N.A	N.A
	Yan [81]	2018	0.7581	0.9846	0.9612	0.9801
	Hu [82]	2018	0.7543	0.9814	0.9632	0.9751
	Oliveira [83]	2018	0.8315	0.9858	0.9694	0.9905
	Alom [84]	2018	0.8298	0.9862	0.9712	0.9914
	Li [85]	2019	0.8465	N.A	0.96733	0.9206
	Jiang [86]	2019	0.8249	0.9904	0.9781	0.9927
**Unsupervised methods**	Zhang [77]	2016	0.7791	0.9758	**0.9554**	0.9748
	Karn [87]	2018	0.80	0.96	**0.96**	0.88
	Aguiree [88]	2018	0.7116	0.9454	0.9231	N.A
	Khan [89]	2018	0.790	0.965	0.951	0.878
	Hashemzadeh [90]	2019	**0.8087**	**0.9892**	**0.9691**	0.9853
	**Proposed (FRANGI)**	2019	**0.8056**	0.9649	0.9526	0.9645
	**Proposed (MULTISCALE)**	2019	**0.8288**	**0.9653**	0.9541	0.9597

**Table 5:** Average time for processing an image.

Type	Methods	Year	Average Time
**Supervised methods**	Aslani [91]	2016	60 s
	Yan [92]	2018	24.79 s
	Hu [82]	2018	1.1 s
	Jiang [86]	2019	2.1 s
**Unsupervised methods**	Khan [93]	2016	1.56 s
	Rodrigues [94]	2017	35 s
	Neto [95]	2017	2.37 s
	Khan [89]	2018	4.56 s
	**Proposed method**	2019	2.70 s

## Data Availability

Datasets used in this study are publicly available on following links: DRIVE: https://drive.grand-challenge.org; STARE: https://cecas.clemson.edu/~ahoover/stare/probing/index.html; CHASE: https://blogs.kingston.ac.uk/retinal/chasedb1; DRIONS: https://www.idiap.ch/software/bob/docs/bob/bob.db.drionsdb/master/index.html.

## References

[1] Fercher A.F., Drexler W., Hitzenberger C.K., Lasser T. (2003). Optical coherence tomography-principles and applications. Rep. Prog. Phys..

[2] Wang M., Jin Q., Wang H., Li D., Baniasadi N., Elze T. (2018). The interrelationship between refractive error, blood vessel anatomy, and glaucomatous visual field loss. Transl. Vis. Sci. Technol..

[3] Elze T., Baniasadi N., Jin Q., Wang H., Wang M. (2017). Ametropia, retinal anatomy, and OCT abnormality patterns in glaucoma. 1. Impacts of refractive error and interartery angle. J. Biomed. Opt..

[4] Brezinski M.E., Tearney G.J., Bouma B.E., Boppart S.A., Hee M.R., Swanson E.A., Southern J.F., Fujimoto J.G. (1996). Imaging of coronary artery microstructure (in vitro) with optical coherence tomography. Am. J. Cardiol..

[5] Williams G.A., Scott I.U., Haller J.A., Maguire A.M., Marcus D., McDonald H.R. (2004). Single-field fundus photography for diabetic retinopathy screening: A report by the American Academy of Ophthalmology. Ophthalmology.

[6] Ng E., Acharya U.R., Rangayyan R.M., Suri J.S. (2014). Ophthalmological Imaging and Applications.

[7] Kolb H. (2003). How the retina works: Much of the construction of an image takes place in the retina itself through the use of specialized neural circuits. Am. Sci..

[8] Keeler C.R. (2002). The ophthalmoscope in the lifetime of Hermann von Helmholtz. Arch. Ophthalmol..

[9] Huang D., Swanson E.A., Lin C.P., Schuman J.S., Stinson W.G., Chang W., Hee M.R., Flotte T., Gregory K., Puliafito C.A. (1991). Optical coherence tomography. Science.

[10] Kolb H. (2011). Simple Anatomy of the Retina by Helga Kolb. Webvision: The Organization of the Retina and Visual System. https://webvision.med.utah.edu/.

[11] Quigley H.A., Broman A.T. (2006). The number of people with glaucoma worldwide in 2010 and 2020. Br. J. Ophthalmol..

[12] Hou Y. (2014). Automatic segmentation of retinal blood vessels based on improved multiscale line detection. J. Comput. Sci. Eng..

[13] Nguyen U.T., Bhuiyan A., Park L.A., Ramamohanarao K. (2013). An effective retinal blood vessel segmentation method using multi-scale line detection. Pattern Recognit..

[14] Perona P., Malik J. (1990). Scale-space and edge detection using anisotropic diffusion. IEEE Trans. Pattern Anal. Mach. Intell..

[15] Yu Y., Acton S.T. (2002). Speckle reducing anisotropic diffusion. IEEE Trans. Image Process..

[16] Krissian K. (2002). Flux-based anisotropic diffusion applied to enhancement of 3-D angiogram. IEEE Trans. Med Imaging.

[17] Aja-Fernández S., Vegas-Sánchez-Ferrero G., Martín-Fernández M., Alberola-López C. (2009). Automatic noise estimation in images using local statistics. Additive and multiplicative cases. Image Vis. Comput..

[18] Portilla J., Strela V., Wainwright M.J., Simoncelli E.P. (2003). Image denoising using scale mixtures of Gaussians in the wavelet domain. IEEE Trans. Image Process..

[19] Dai M., Peng C., Chan A.K., Loguinov D. (2004). Bayesian wavelet shrinkage with edge detection for SAR image despeckling. IEEE Trans. Geosci. Remote Sens..

[20] Blu T., Luisier F. (2007). The SURE-LET approach to image denoising. IEEE Trans. Image Process..

[21] Remenyi N., Nicolis O., Nason G., Vidakovic B. (2014). Image denoising with 2D scale-mixing complex wavelet transforms. IEEE Trans. Image Process..

[22] Ur Rehman N., Naveed K., Ehsan S., McDonald-Maier K. Multi-scale image denoising based on goodness of fit (GOF) tests. Proceedings of the 2016 24th European Signal Processing Conference (EUSIPCO).

[23] Naveed K., Shaukat B., Ehsan S., Mcdonald-Maier K.D., ur Rehman N. (2019). Multiscale image denoising using goodness-of-fit test based on EDF statistics. PLoS ONE.

[24] Naveed K., Ehsan S., McDonald-Maier K.D., Rehman N.U. (2019). A Multiscale Denoising Framework Using Detection Theory with Application to Images from CMOS/CCD Sensors. Sensors.

[25] Buades A., Coll B., Morel J.M. A non-local algorithm for image denoising. Proceedings of the 2005 IEEE Computer Society Conference on Computer Vision and Pattern Recognition (CVPR’05).

[26] Deledalle C.A., Denis L., Tupin F. (2009). Iterative weighted maximum likelihood denoising with probabilistic patch-based weights. IEEE Trans. Image Process..

[27] Coupé P., Hellier P., Kervrann C., Barillot C. Bayesian non local means-based speckle filtering. Proceedings of the 2008 5th IEEE International Symposium on Biomedical Imaging: From Nano to Macro.

[28] Di Martino G., Di Simone A., Iodice A., Riccio D. (2016). Scattering-based nonlocal means SAR despeckling. IEEE Trans. Geosci. Remote Sens..

[29] Dabov K., Foi A., Katkovnik V., Egiazarian K. (2007). Image denoising by sparse 3-D transform-domain collaborative filtering. IEEE Trans. Image Process..

[30] Parrilli S., Poderico M., Angelino C.V., Verdoliva L. (2011). A nonlocal SAR image denoising algorithm based on LLMMSE wavelet shrinkage. IEEE Trans. Geosci. Remote Sens..

[31] Jamal I., Akram M.U., Tariq A. (2012). Retinal image preprocessing: Background and noise segmentation. Telkomnika.

[32] Hani A.F.M., Soomro T.A., Fayee I., Kamel N., Yahya N. Identification of noise in the fundus images. Proceedings of the 2013 IEEE International Conference on Control System, Computing and Engineering Penang.

[33] Dai P., Sheng H., Zhang J., Li L., Wu J., Fan M. (2016). Retinal fundus image enhancement using the normalized convolution and noise removing. Int. J. Biomed. Imaging.

[34] Khawaja A., Khan T.M., Naveed K., Naqvi S.S., Rehman N.U., Nawaz S.J. (2019). An Improved Retinal Vessel Segmentation Framework Using Frangi Filter Coupled with the Probabilistic Patch Based Denoiser. IEEE Access.

[35] Coifman R.R., Donoho D.L. (1995). Translation-invariant de-noising. Wavelets and Statistics.

[36] ur Rehman N., Abbas S.Z., Asif A., Javed A., Naveed K., Mandic D.P. (2017). Translation invariant multi-scale signal denoising based on goodness-of-fit tests. Signal Process..

[37] Naveed K., ur Rehman N. (2020). Wavelet based multivariate signal denoising using Mahalanobis distance and EDF statistics. IEEE Trans. Signal Process..

[38] Witkin A. (1984). Scale-space filtering: A new approach to multi-scale description. Proceedings of the ICASSP’84, IEEE International Conference on AcousticsSpeech, and Signal Processing.

[39] Shin D.H., Park R.H., Yang S., Jung J.H. (2005). Block-based noise estimation using adaptive Gaussian filtering. IEEE Trans. Consum. Electron..

[40] Babaud J., Witkin A.P., Baudin M., Duda R.O. (1986). Uniqueness of the Gaussian kernel for scale-space filtering. IEEE Trans. Pattern Anal. Mach. Intell..

[41] Deng G., Cahill L. (1993). An adaptive Gaussian filter for noise reduction and edge detection. Proceedings of the 1993 IEEE Conference Record Nuclear Science Symposium and Medical Imaging Conference.

[42] Tomasi C., Manduchi R. Bilateral filtering for gray and color images. Proceedings of the ICCV.

[43] Farbman Z., Fattal R., Lischinski D., Szeliski R. (2008). Edge-preserving decompositions for multi-scale tone and detail manipulation. ACM Transactions on Graphics (TOG).

[44] Fattal R., Agrawala M., Rusinkiewicz S. (2007). Multiscale shape and detail enhancement from multi-light image collections. ACM Transactions on Graphics (TOG).

[45] Kang H., Lee S., Chui C.K. (2008). Flow-based image abstraction. IEEE Trans. Vis. Comput. Graph..

[46] Chen J., Paris S., Durand F. (2007). Real-time edge-aware image processing with the bilateral grid. ACM Transactions on Graphics (TOG).

[47] Xiao J., Cheng H., Sawhney H., Rao C., Isnardi M. (2006). Bilateral filtering-based optical flow estimation with occlusion detection. European Conference on Computer Vision.

[48] Sun D., Roth S., Black M.J. Secrets of optical flow estimation and their principles. Proceedings of the 2010 IEEE Computer Society Conference on Computer Vision and Pattern Recognition.

[49] Lalli G., Kalamani D., Manikandaprabu N., Brindha S. (2013). Features Recognition on Retinal Fundus Image—A Multi-Systemic Comparative Analysis. Int. J. Adv. Res. Comput. Sci. Softw. Eng..

[50] Manduca A., Yu L., Trzasko J.D., Khaylova N., Kofler J.M., McCollough C.M., Fletcher J.G. (2009). Projection space denoising with bilateral filtering and CT noise modeling for dose reduction in CT. Med. Phys..

[51] Anand C.S., Sahambi J. (2008). MRI denoising using bilateral filter in redundant wavelet domain. TENCON 2008–2008 IEEE Region 10 Conference.

[52] Shi F., Chen X., Zhao H., Zhu W., Xiang D., Gao E., Sonka M., Chen H. (2014). Automated 3-D retinal layer segmentation of macular optical coherence tomography images with serous pigment epithelial detachments. IEEE Trans. Med. Imaging.

[53] Katz N., Nelson M., Goldbaum M., Chaudhuri S., Chatterjee S. (1989). Detection of blood vessels in retinal images using two-dimensional matched filters. IEEE Trans. Med. Imaging.

[54] Hoover A., Kouznetsova V., Goldbaum M. (1998). Locating blood vessels in retinal images by piece-wise threshold probing of a matched filter response. Proceedings of the AMIA Symposium.

[55] Zhang B., Zhang L., Zhang L., Karray F. (2010). Retinal vessel extraction by matched filter with first-order derivative of Gaussian. Comput. Biol. Med..

[56] Aylward S., Pizer S., Bullitt E., Eberly D. Intensity ridge and widths for tubular object segmentation and description. Proceedings of the IEEE Workshop on Mathematical Methods in Biomedical Image Analysis.

[57] Eberly D., Gardner R., Morse B., Pizer S., Scharlach C. (1994). Ridges for image analysis. J. Math. Imaging Vis..

[58] Soares J.V., Leandro J.J., Cesar R.M., Jelinek H.F., Cree M.J. (2006). Retinal vessel segmentation using the 2-D Gabor wavelet and supervised classification. IEEE Trans. Med. Imaging.

[59] Ricci E., Perfetti R. (2007). Retinal blood vessel segmentation using line operators and support vector classification. IEEE Trans. Med. Imaging.

[60] Lorenz C., Carlsen I.C., Buzug T.M., Fassnacht C., Weese J. (1997). A multi-scale line filter with automatic scale selection based on the Hessian matrix for medical image segmentation. International Conference on Scale-Space Theories in Computer Vision.

[61] Frangi A.F., Niessen W.J., Vincken K.L., Viergever M.A. (1998). Multiscale vessel enhancement filtering. International Conference on Medical Image Computing and Computer-Assisted Intervention.

[62] Rossmann K. (1969). Point spread-function, line spread-function, and modulation transfer function: Tools for the study of imaging systems. Radiology.

[63] Manzanares A., Calvo M., Chevalier M., Lakshminarayanan V. (1997). Line spread function formulation proposed by WH Steel: A revision. Appl. Opt..

[64] Williams C.S., Becklund O.A. (1989). Introduction to the Optical Transfer Function.

[65] Nicolai M., Franceschi A., Turris S., Rosati A., Pirani V., Mariotti C. (2019). Papillary Vessel Density Changes After Intravitreal Anti-VEGF Injections in Hypertensive Patients with Central Retinal Vein Occlusion: An Angio-OCT Study. J. Clin. Med..

[66] Arsalan M., Owais M., Mahmood T., Cho S.W., Park K.R. (2019). Aiding the Diagnosis of Diabetic and Hypertensive Retinopathy Using Artificial Intelligence-Based Semantic Segmentation. J. Clin. Med..

[67] Arrigo A., Romano F., Albertini G., Aragona E., Bandello F., Battaglia Parodi M. (2019). Vascular Patterns in Retinitis Pigmentosa on Swept-Source Optical Coherence Tomography Angiography. J. Clin. Med..

[68] Palochak C.M.A., Lee H.E., Song J., Geng A., Linsenmeier R.A., Burns S.A., Fawzi A.A. (2019). Retinal Blood Velocity and Flow in Early Diabetes and Diabetic Retinopathy Using Adaptive Optics Scanning Laser Ophthalmoscopy. J. Clin. Med..

[69] Sacconi R., Casaluci M., Borrelli E., Mulinacci G., Lamanna F., Gelormini F., Carnevali A., Querques L., Zerbini G., Bandello F. (2019). Multimodal Imaging Assessment of Vascular and Neurodegenerative Retinal Alterations in Type 1 Diabetic Patients without Fundoscopic Signs of Diabetic Retinopathy. J. Clin. Med..

[70] Lindeberg T. (1998). Feature Detection with Automatic Scale Selection. Int. J. Comput. Vis..

[71] King P., Hubner K., Gibbs W., Holloway E. (1981). Noise identification and removal in positron imaging systems. IEEE Trans. Nucl. Sci..

[72] Zuiderveld K. (1994). Contrast limited adaptive histogram equalization. Graphics Gems IV.

[73] Ridler T., Calvard S. (1978). Picture thresholding using an iterative selection method. IEEE Trans. Syst. Man Cybern..

[74] Feijoo J., de la Casa J., Servet H., Zamorano M., Mayoral M., Suárez E. (2014). DRIONS-DB: Digital Retinal Images for Optic Nerve Segmentation Database. https://www.idiap.ch/software/bob/docs/bob/bob.db.drionsdb/master/index.html.

[75] Hoover A.D., Kouznetsova V., Goldbaum M. (2000). Locating blood vessels in retinal images by piecewise threshold probing of a matched filter response. IEEE Trans. Med. Imaging.

[76] Niemeijer M., Staal J., van Ginneken B., Loog M., Abramoff M.D. (2004). Comparative study of retinal vessel segmentation methods on a new publicly available database. Proc. SPIE.

[77] Zhang J., Dashtbozorg B., Bekkers E., Pluim J.P., Duits R., ter Haar Romeny B.M. (2016). Robust retinal vessel segmentation via locally adaptive derivative frames in orientation scores. IEEE Trans. Med. Imaging.

[78] Li Q., Feng B., Xie L., Liang P., Zhang H., Wang T. (2016). A Cross-Modality Learning Approach for Vessel Segmentation in Retinal Images. IEEE Trans. Med. Imaging.

[79] Orlando J.I., Prokofyeva E., Blaschko M.B. (2016). A discriminatively trained fully connected conditional random field model for blood vessel segmentation in fundus images. IEEE Trans. Biomed. Eng..

[80] Dasgupta A., Singh S. A fully convolutional neural network based structured prediction approach towards the retinal vessel segmentation. Proceedings of the 2017 IEEE 14th International Symposium on Biomedical Imaging (ISBI 2017).

[81] Yan Z., Yang X., Cheng K.T. (2018). Joint segment-level and pixel-wise losses for deep learning based retinal vessel segmentation. IEEE Trans. Biomed. Eng..

[82] Hu K., Zhang Z., Niu X., Zhang Y., Cao C., Xiao F., Gao X. (2018). Retinal vessel segmentation of color fundus images using multiscale convolutional neural network with an improved cross-entropy loss function. Neurocomputing.

[83] Oliveira A., Pereira S., Silva C.A. (2018). Retinal vessel segmentation based on fully convolutional neural networks. Expert Syst. Appl..

[84] Alom M.Z., Hasan M., Yakopcic C., Taha T.M., Asari V.K. (2018). Recurrent residual convolutional neural network based on u-net (r2u-net) for medical image segmentation. arXiv.

[85] Li R., Li M., Li J. (2019). Connection Sensitive Attention U-NET for Accurate Retinal Vessel Segmentation. arXiv.

[86] Jiang Y., Tan N., Peng T., Zhang H. (2019). Retinal Vessels Segmentation Based on Dilated Multi-Scale Convolutional Neural Network. arXiv.

[87] Karn P.K., Biswal B., Samantaray S.R. (2018). Robust retinal blood vessel segmentation using hybrid active contour model. IET Image Process..

[88] Aguirre-Ramos H., Avina-Cervantes J.G., Cruz-Aceves I., Ruiz-Pinales J., Ledesma S. (2018). Blood vessel segmentation in retinal fundus images using Gabor filters, fractional derivatives, and Expectation Maximization. Appl. Math. Comput..

[89] Khan K.B., Khaliq A.A., Jalil A., Shahid M. (2018). A robust technique based on VLM and Frangi filter for retinal vessel extraction and denoising. PLoS ONE.

[90] Hashemzadeh M., Azar B.A. (2019). Retinal Blood Vessel Extraction Employing Effective Image Features and Combination of Supervised and Unsupervised Machine Learning Methods. Artif. Intell. Med..

[91] Aslani S., Sarnel H. (2016). A new supervised retinal vessel segmentation method based on robust hybrid features. Biomed. Signal Process. Control.

[92] Yan Z., Yang X., Cheng K.T. (2017). A skeletal similarity metric for quality evaluation of retinal vessel segmentation. IEEE Trans. Med. Imaging.

[93] BahadarKhan K., Khaliq A.A., Shahid M. (2016). A morphological hessian based approach for retinal blood vessels segmentation and denoising using region based otsu thresholding. PLoS ONE.

[94] Rodrigues L.C., Marengoni M. (2017). Segmentation of optic disc and blood vessels in retinal images using wavelets, mathematical morphology and Hessian-based multi-scale filtering. Biomed. Signal Process. Control.

[95] Neto L.C., Ramalho G.L., Neto J.F.R., Veras R.M., Medeiros F.N. (2017). An unsupervised coarse-to-fine algorithm for blood vessel segmentation in fundus images. Expert Syst. Appl..

